# Amyloid Beta monomers regulate cyclic adenosine monophosphate response element binding protein functions by activating type‐1 insulin‐like growth factor receptors in neuronal cells

**DOI:** 10.1111/acel.12684

**Published:** 2017-11-01

**Authors:** Stefania Zimbone, Irene Monaco, Fiorenza Gianì, Giuseppe Pandini, Agata G. Copani, Maria Laura Giuffrida, Enrico Rizzarelli

**Affiliations:** ^1^ Institute of Biostructures and Bioimaging National Council of Research (IBB‐CNR) Via Paolo Gaifami 18 95126 Catania Italy; ^2^ Endocrinology, Department of Clinical and Experimental Medicine Garibaldi‐Nesima Medical Center University of Catania via Palermo 636 95122 Catania Italy; ^3^ Department of Drug Sciences University of Catania Viale A. Doria 6 95125 Catania Italy; ^4^ Department of Chemical Sciences University of Catania Viale A. Doria 6 95125 Catania Italy

**Keywords:** Aβ monomers, Alzheimer's disease, BDNF, CREB, IGF1‐R

## Abstract

Alzheimer's disease (AD) is a progressive neurodegenerative disorder associated with synaptic dysfunction, pathological accumulation of β‐amyloid (Aβ), and neuronal loss. The self‐association of Aβ monomers into soluble oligomers seems to be crucial for the development of neurotoxicity (*J. Neurochem*., 00, 2007 and 1172). Aβ oligomers have been suggested to compromise neuronal functions in AD by reducing the expression levels of the CREB target gene and brain‐derived neurotrophic factor (BDNF) (*J. Neurosci*., 27, 2007 and 2628; Neurobiol. Aging, 36, 2015 and 20406 *Mol. Neurodegener*., 6, 2011 and 60). We previously reported a broad neuroprotective activity of physiological Aβ monomers, involving the activation of type‐1 insulin‐like growth factor receptors (IGF‐IRs) (*J. Neurosci*., 29, 2009 and 10582, *Front Cell Neurosci*., 9, 2015 and 297). We now provide evidence that Aβ monomers, by activating the IGF‐IR‐stimulated PI3‐K/AKT pathway, induce the activation of CREB in neurons and sustain BDNF transcription and release.

**Abbreviations**
AD: Alzheimer's disease
AKT: BDNF, brain‐derived neurotrophic factor
APP: amyloid precursor protein
Ara‐C: cytosine arabinoside (1‐β‐D‐arabinofuranosylcytosine
Aβ: Amyloid beta
CREB: cyclic adenosine monophosphate response element binding protein
CRE: cAMP response elements
GSK3‐β: glycogen synthase kinase‐3 beta
IGF‐IRs: type‐1 insulin‐like growth factor receptors
MCI: mild cognitive impairment
nNOS: neuronal nitric oxide synthase
PFC: postmortem prefrontal cortex
PI‐3‐K/AKT: phosphatidylinositol 3‐kinase/AKT
PPP: picropodophyllin

## Introduction

Alzheimer's disease (AD) is the most common form of dementia in the elderly. The onset of the disease starts a decade or more before the first signs of cognitive impairment appear. For this reason, a great attention is being paid to the early events occurring in the AD brain, such as synaptic dysfunctions (Masliah *et al*., 2001).

Pathological accumulation of β‐amyloid (Aβ) is the feature of AD (Hardy & Allsop, 1991; Selkoe, 1991; Hardy & Higgins, 1992). Specifically, soluble Aβ oligomers are fundamental to promote neurotoxicity (Klein, 2001; Walsh & Selkoe, 2007) through different ways (Ferreira & Klein, 2011; Pearson‐Leary & McNay, 2012), including altered levels of neurotrophic factors (NTFs) (Calissano *et al*., 2010; Budni *et al*., 2015). Among these, brain‐derived neurotrophic factor (BDNF) (Barde *et al*., 1982) is essential in sustaining physiological processes of the normal adult brain (Nagahara & Tuszynski, 2011), by tuning: (i) dendritic branching and spine morphology (Horch & Katz, 2002) and (ii) synaptic plasticity and long‐term potentiation (Figurov *et al*., 1996) and therefore learning and memory. Interestingly, postmortem studies have shown that BDNF mRNA and protein decrease not only in end‐stage disease, but also in patients diagnosed with mild cognitive impairment (MCI) (Peng *et al*., 2005), suggesting that a BDNF loss could be involved in the early synaptic dysfunctions.

The cyclic adenosine monophosphate response element binding protein (CREB) mediates the transcription of BDNF. CREB is activated in neurons in response to trans‐synaptic signaling and regulates the expression of genes that are essential for adaptive neuronal responses, as well as more complex neural functions, such as learning and memory (Alberini & Chen, 2012). CREB target genes include immediate‐early genes, such as c‐fos (Sheng *et al*., 1990), and others important for synaptic function, such as BDNF (Shieh *et al*., 1998) and neuronal nitric oxide synthase (nNOS) (Sasaki *et al*., 2000). CREB activation is induced through ser‐133 phosphorylation mediated by a variety of kinases, including the PI3K‐activated AKT (reviewed in Lonze & Ginty, 2002). The PI3K/AKT pathway, by inhibiting glycogen synthase kinase‐3 beta (GSK3‐β), could also release CREB‐mediated transcription from the blockade operated by GSK3‐β phosphorylation at ser‐129 (Grimes & Jope, 2001; Zheng & Quirion, 2006).

We have previously reported that, differently from Aβ oligomers, Aβ monomers (i.e., the native physiological form of Aβ) are devoid of intrinsic toxicity, are endowed, instead, with a broad neuroprotective activity (Giuffrida *et al*., 2009), and support neuronal glucose uptake by acting as positive allosteric modulators of type‐1 insulin‐like growth factor receptors (IGF‐IR) (Giuffrida *et al*., 2015). By mimicking IGF1 signaling, Aβ monomers activate the PI3K/AKT pathway, with ensuing ser‐9 phosphorylation (inhibition) of the AKT substrate, GSK‐3β (Giuffrida *et al*., 2009).

Here we demonstrate that Aβ monomers, both synthetic and natural, by activating PI3K/AKT pathway, were able to induce CREB activation in differentiated human neuroblastoma cells and in primary rat cortical neurons. This effect was not shared by pathogenic Aβ oligomers. Accordingly, CREB activation is impaired in the brain of patients with AD and Tg2576 mice (Pugazhenthi *et al*., 2011; Bartolotti *et al*., 2016a,b), where it is associated with decreased levels of CREB‐regulated BDNF release (Pugazhenthi *et al*., 2011). Contrary to what takes place in the disease, we found that CREB activation induced by Aβ monomers was paralleled by an increased neuronal BDNF release.

## Results

### Aβ(1‐42) monomers induce CREB phosphorylation in primary cortical neurons and in differentiated neuroblastoma cells

**Figure 1:**
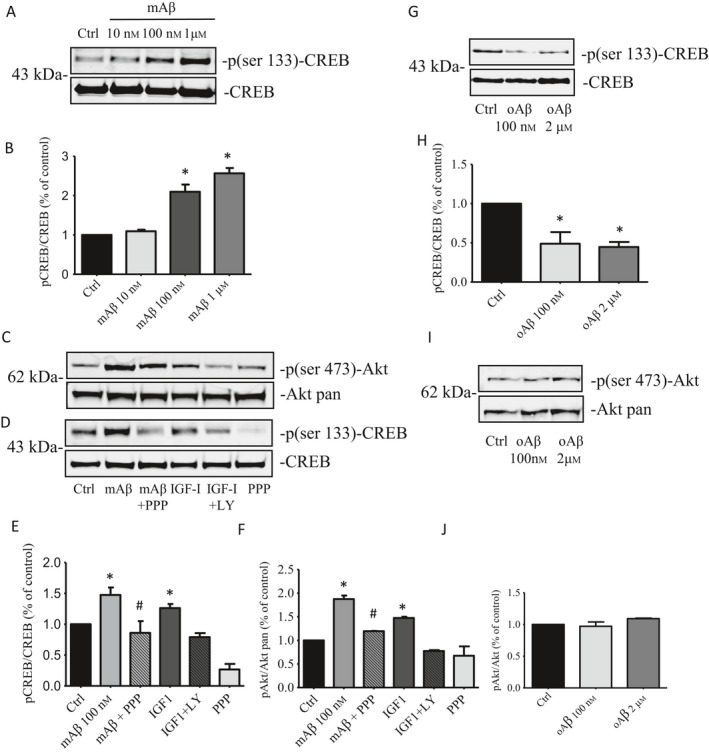
Effects of Aβ monomers on pCREB and pAKT expression levels. (A) Representative immunoblot showing that a 30’ exposure of cortical neurons to increasing concentrations of monomers was able to induce a concentration‐dependent phosphorylation of CREB protein. (B) The band intensities of pCREB were quantified, after normalization to the total CREB, from three separate Western blots. Bar values are expressed as % of the respective controls. *P < 0.05 vs. CTRL by one‐way ANOVA + Tukey's test. Representative immunoblot of pAkt (C) and pCREB (D) bands in untreated cultures, and cultures treated with mAβ for 30’. Total Akt and total CREB bands are shown for control of loading. Treatments with the PI3K inhibitor, LY294002 (10 μm), or the selective inhibitor of IGF‐IR, picropodophyllin (PPP, 500 nm), reduced the expression of both pAKT (ser‐473) and pCREB (ser‐133), which were induced by 100 nm mAβ. Densitometric analyzes of three separate Western blots, in which pAkt (E) and pCREB (F) signals were normalized to the respective totals, are displayed. Values are expressed as % of the respective controls. *P < 0.05 vs. CTRL by one‐way ANOVA +Tukey's test. #*P* < 0.05 vs. mAβ by one‐way ANOVA + Tukey's test. (G) Representative immunoblot of pCREB levels following a 30’ exposure of cortical neurons to Aβ oligomers (oAβ 100 nm or 2 μm). (H) Normalized densitometric values of pCREB, following oAβ exposure, are expressed as % of the respective controls and derive from three separate Western blots. *P < 0.05 vs. CTRL by one‐way densitometric + Tukey's test. (I) Representative immunoblot of pAkt levels following 30’ exposure to 100 nm or 2 μm oAβ. (J) Normalized densitometric values of pAkt, following oAβ exposure, are expressed as % of the respective controls and derive from three separate Western blots.

To determine whether Aβ monomers (mAβ) were able to promote the activation of CREB, we assessed the expression levels of p(ser133)‐CREB in mature cultures of pure cortical neurons. A 30‐min exposure was sufficient for mAβ to induce p(ser133)‐CREB expression in a dose‐dependent manner (0.01–1 μm) (Fig. 1A,B), as assessed by Western blot analysis. Consistent with the ability of mAβ to activate the PI3K pathway by engaging IGF‐IRs (Giuffrida *et al*., 2009, 2015), the selective inhibitor of IGF‐IR, picropodophyllin (PPP, 500 nm), and the PI3K inhibitor, LY294002 (10 μm), reduced the phosphorylation of both p(ser473)‐AKT (Fig. 1C,E) and p(ser‐133)CREB (Fig. 1D,F), which were induced by 100 nm mAβ. Unlike mAβ, Aβ oligomers (oAβ) reduced the basal expression of p(ser‐133)CREB (Fig. 1G,H) at a concentration as low as 100 nm, leaving unaffected basal levels of p(ser473)‐AKT up to a 2 μm concentration (Fig. 1I,L). Appropriate concentrations of oAβ for signaling studies were selected based on parallel assessment of neuronal viability by MTT assay. Both concentrations of oAβ were devoid of toxicity at 24h, and toxic to a different extent after 48 hours exposure (% neuronal survival: controls = 100 ± 10.33; oAβ 100 nm = 82.3 ± 5.03; oAβ 2 μm = 67.93 ± 3.52).

**Figure 2:**
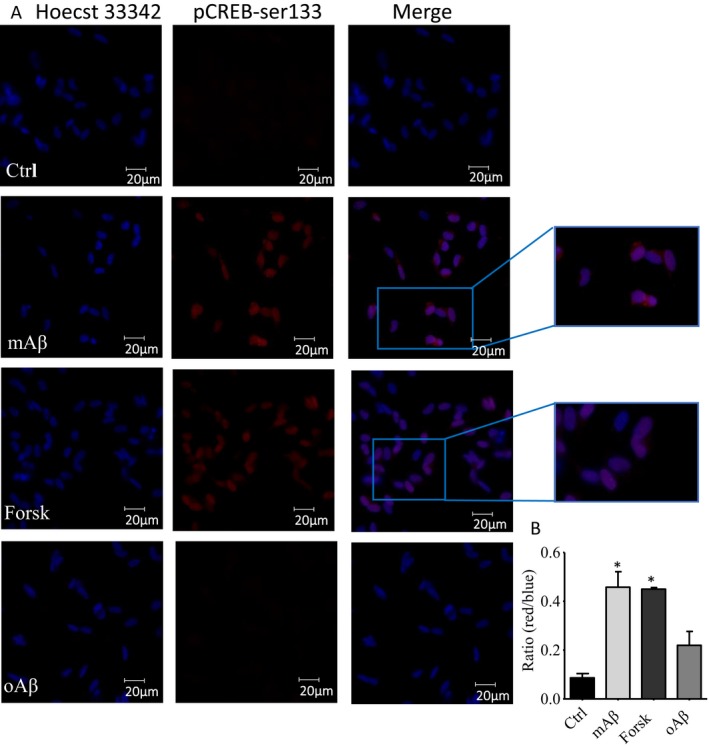
Effect of Aβ monomers on nuclear localization of pCREB. (A) Immunofluorescence staining for p(ser133)‐CREB (red) in differentiated SH‐SY5Y after 30’ incubation with either 100 nm Aβ monomers or oligomers, or 30 μm forskolin. Cell nuclei were counterstained with Hoechst 33342 (blue). (B) The nuclear staining of pCREB was quantified by measuring the ratio between the red and the blue signal. Bars are means ± SEM (*n* = 60) of red/blue ratio values within 20 nuclear ROI/field, in 3 fields/dish. Significantly different from Ctrl (*) at *P* < 0.05 (one‐way ANOVA + Tukey's test).

We also assessed CREB phosphorylation in the differentiated neuroblastoma SH‐SY5Y, exposed for 30 min to 100 nm of either mAβ or oAβ. Changes in CREB phosphorylation were assessed by p(ser‐133)‐CREB immunofluorescent staining of cell nuclei, which were counterstained with Hoechst dye 33342 (1 μg μL^−1^). Consistent with its transcriptional activity, p(ser‐133)‐CREB was largely imaged within the cell nuclei following exposure to mAβ (100 nm). The same nuclear pattern was observed by treating the cells with forskolin (30 μm), which was used as a positive control for imaging CREB activation, but not with synthetic Aβ oligomers (oAβ, 100 nm) (Fig. 2A,B).

### mAβ induces CREB DNA binding activity in cortical neurons

**Figure 3:**
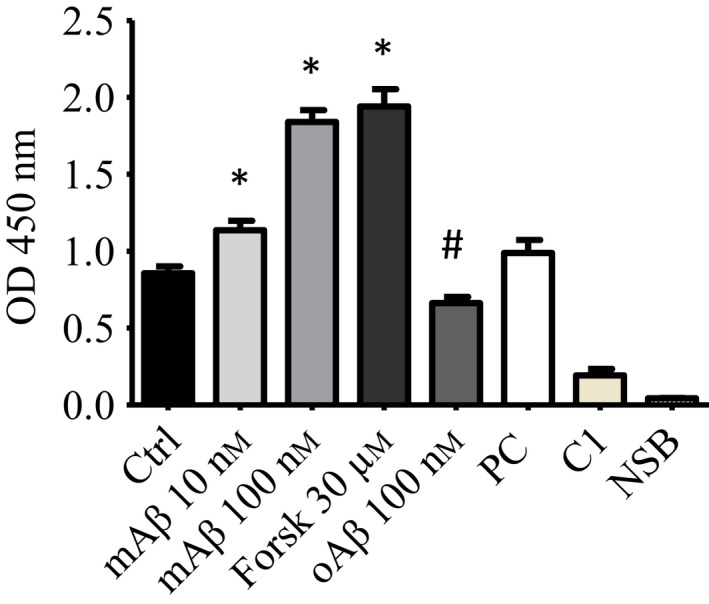
Effect of Aβ monomers on CREB DNA binding activity. Neurons were treated with two different concentrations of mAβ (10 and 100 nm). 100 nm Aβ oligomers (oAβ) and 30 μm Forskolin were used as negative and positive control, respectively. Whole cell lysates of pure neuronal cultures were tested with Transcription factor Assay kit. Bars are means of three independent experiments (*n* = 5). Significantly different from Ctrl (*) or from mAβ 100 nm condition (#) at *P* < 0.05 (one‐way ANOVA + Tukey's test). PC (clarified cell lysates), C1 (competitor dsDNA), and NSB (Nonspecific Binding wells) bars represent standard controls provided by the assay kit.

To assess whether mAβ activates CREB to induce the transcription of target genes, whole neuronal lysates were used to assay CREB binding to immobilized CRE (cAMP response elements) consensus sequences. Consistent with its ability to induce p(ser‐133)‐CREB, 100 nm mAβ for 30 min promoted a twofold increase in the spectrophotometric signal detecting the activated CREB/CRE transcription factor complex (Fig 3). A similar increase was observed with 30 μm forskolin, used as positive control for the experiment, whereas 100 nm oAβ failed to induce the transactivating activity of CREB (Fig. 3).

### Native forms of Aβ from H4‐APPswe cells induce pCREB(ser‐133) in primary cortical neurons

**Figure 4:**
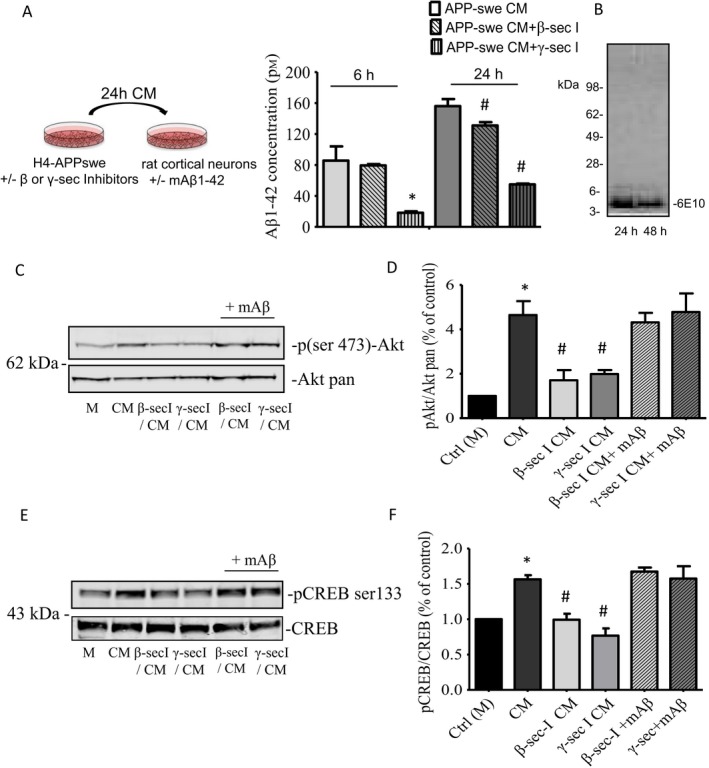
Effect of native Aβ monomers on CREB phosphorylation. (A) ELISA determination of Aβ42 levels in conditioned medium (CM) from H4‐APPswe neuroglioma cells after a 24‐h incubation in the presence or absence of β‐ and γ‐secretases inhibitors (as described in the schematic representation). Bars are means ± SEM of two independent experiments (each condition was run three times within each experiment). Significantly different from Ctrl 6h (*) or from Ctrl 24h (#) at *P* < 0.05 (one‐way ANOVA + Tukey's test). (B) 24‐ and 48h conditioned media from H4‐APPswe (5 μL) were analyzed by Western blot using the 6E10 antibody. The image shows a single 4 kD band, corresponding to the molecular weight of Aβ monomer. Representative immunoblots of pAkt (C) and pCREB (E) in untreated cultures, and cultures treated for 30 min with the conditioned medium (CM) from H4‐APPswe cells. Native Aβ from CM increased both pCREB (ser‐133) and pAkt (ser473). CM collected in the presence of either β‐ or γ‐secretase inhibitors prevented the increase of pCREB and pAkt expression. Exogenous addition of synthetic mAβ to the secretase inhibitor‐treated CM was able to rescue the levels of the phosphorylated forms of CREB and Akt. The band intensities of pAkt (D) were quantified after normalization to the total Akt in two separate Western blots. Values (means ± SD) are expressed as % of the respective controls. *P < 0.05 vs. CTRL (M) by one‐way ANOVA + Tukey's test. #*P* < 0.05 vs. CM by one‐way ANOVA + Tukey's test. The band intensities of pCREB (F) were quantified after normalization to the total CREB in three separate Western blots. Values (means ± SEM) are expressed as % of the respective controls. *P < 0.05 vs. CTRL (M) by one‐way ANOVA + Tukey's test. #*P* < 0.05 vs. CM by one‐way ANOVA + Tukey's test.

We wondered whether native forms of Aβ were able to replicate the effects of synthetic Aβ monomers. To answer this question, we used the H4‐APPswe cell line, engineered to over‐produce Aβ1–40 (Aβ40) and Aβ1–42 (Aβ42) by the introduction of the double Swedish mutation (K595N/M596L) in the amyloid precursor protein (APP) gene. Aβ levels reached in the conditioned medium (CM) of this stable transfected neuroglioma are low, likely insufficient to induce significant aggregation of the peptide (Lomakin *et al*., 1997; Harper *et al*., 1999). We collected a 6h and 24h conditioned medium from H4‐APPswe cultures, incubated in presence or absence of β‐ or γ‐secretase inhibitors, and Aβ levels were measured by ELISA (Fig. 4A). Data showed that, after 24h, Aβ concentration in the conditioned medium reached the range of few hundred picomolars, and it was sensitive to the presence of the secretase inhibitors (Fig. 4A). The 6E10 monoclonal antibody, raised against the 1–16 fragment of Aβ, failed to reveal amyloid bands other than the 4kD monomer in the neuroglioma medium even when collected after 48h (Fig. 4B). Hence, we used the 24h conditioned medium to treat cortical neurons, and we evaluated the activation of CREB after 30‐min exposure. Unconditioned medium was used as control. As expected, we observed an increased expression of both p(ser473)‐AKT and p(ser‐133)CREB after exposing neurons to H4‐APPswe CM, which was prevented in the presence of either β‐ or γ‐secretase inhibitor‐treated CM (Fig. 4C–F). Interestingly, the exogenous addition of synthetic mAβ to the secretase inhibitor‐treated CM was able to rescue the levels of both p(ser473)‐AKT and p(ser‐133)‐CREB (Fig. 4C–F).

### mAβ promotes the transcription and release of the CREB target gene, BDNF, in primary cortical neurons

**Figure 5:**
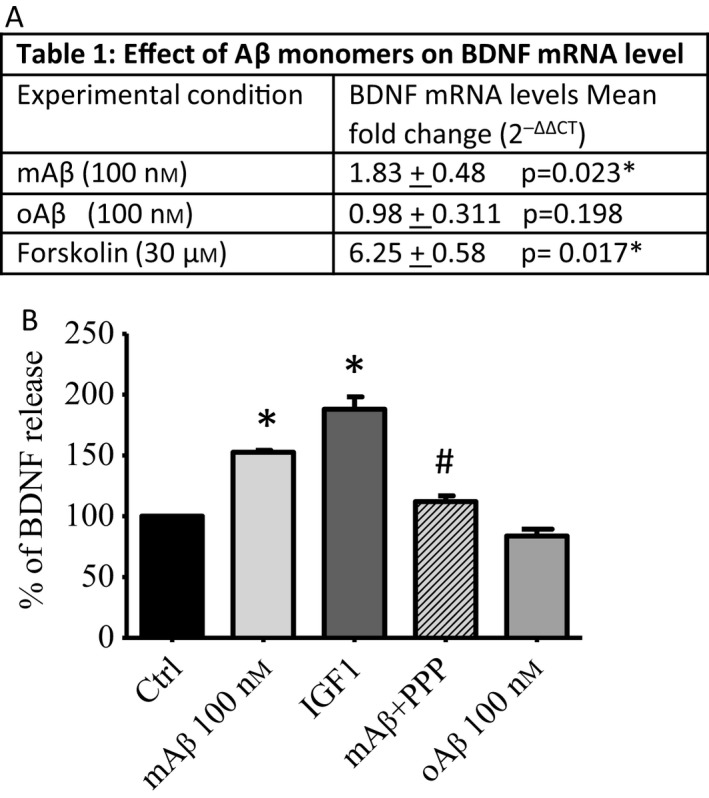
Effect of Aβ monomers on BDNF transcription and release. (A) Data represent mean fold changes + SEM with respect to the control condition. Two technical replicates/condition in five separate real‐time PCR experiments were run following 24h exposure to either 100 nm mAβ or oAβ. Forskolin (30 μm) was used as a positive control. (B) BDNF release as measured by ELISA assay at 24h. Pure cortical neurons were treated with 100 nm mAβ, in the presence or absence of picropodophyllin (PPP), or 100 nm oAβ. IGF‐1 (5 ng/ml) was used as a positive control. Values are expressed as % of control. Bars are means ± SEM of two to three independent experiments (*n* = 4). *P < 0.05 vs. Ctrl and #*P* < 0.05 vs. mAβ.

Mature cortical neurons were treated with 100 nm mAβ or 100 nm oAβ for 24h, and the transcript for BDNF was quantified by real‐time PCR. Once more, 30 μm forskolin was used as a positive control. mAβ induced a discreet and consistent increase in BDNF mRNA levels across the experiments (Fig. 5A), and the heightened BDNF transcription was accompanied by an increase in neuronal BDNF release, as assessed by ELISA (Fig. 5B). As in the case of p(ser‐133)‐CREB expression, the IGF‐IR inhibitor, PPP, prevented the activity of mAβ, which was instead mimicked by IGF1 (5 ng mL^−1^) (Fig. 5B). Unlike mAβ, oAβ did not affect BDNF transcription (Fig. 5A) and release (Fig. 5B).

## Discussion

Growing evidence exists that native Aβ, which is produced during neuronal activity (Cirrito *et al*., 2005), may subserve physiological functions (reviewed in Giuffrida *et al*., 2010; Kepp, 2016). In particular, endogenous Aβ seems to be relevant for the control of synaptic activity and memory formation (Puzzo *et al*., 2011; Piccini *et al*., 2012), via the activation of α‐7‐nAChRs (Calabrese, 2001). Based on this evidence, Teich *et al*. (2015) have suggested a central role for CREB and associated memory‐related genes in the synaptic effects of native Aβ.

Here, we provide the first evidence that Aβ, in its monomeric form, induces the activation of CREB in neurons and promotes the transcription and the release of the CREB target gene, BDNF. Specifically, a 30‐min stimulation of primary cortical neurons with synthetic mAβ (100 nm) resulted in phosphorylation of CREB at serine 133 (p(ser‐133)CREB). This residue is a key site that must be phosphorylated for CREB to function as transcriptional regulator (Lonze & Ginty, 2002). Accordingly, mAβ‐activated neuronal CREB bound to CRE consensus sequences, and a nuclear localization of p(ser‐133)‐CREB was found in differentiated SH‐SY5Y cells briefly exposed to mAβ.

Many routes converge into CREB phosphorylation (Lonze & Ginty, 2002), including the well‐documented cAMP‐PKA pathway. Indeed, forskolin, a drug that raises cAMP levels, was able to increase p(ser‐133)‐CREB in SH‐SY5Y cells. However, at least under some circumstances, the PI3K/AKT pathway appears to control CREB phosphorylation in neurons (Zheng & Quirion, 2006). Consistent with our previous demonstration that mAβ activates PI3K/AKT signaling by functioning as a positive allosteric modulator of IGF‐IR (Giuffrida *et al*., 2009, 2015), both the specific IGF‐IR inhibitor, PPP, and the PI3K inhibitor, LY294002, reduced strongly the activation of CREB promoted by mAβ in cortical neurons.

The evidence that native human Aβ monomers, from the conditioned media of neuroglioma cells, induced CREB activation in neurons strengthened results obtained with synthetic Aβ monomers. Aβ peptides from different sources (i.e., cell‐secreted, brain‐derived or synthetic) are significantly different in terms of isoforms and effective concentrations (Reed *et al*., 2011).

Picomolar concentrations of a synthetic Aβ solution, containing both monomers and oligomers, have been shown to mimic the memory enhancement effects of endogenous Aβ (Puzzo *et al*., 2008). Instead, the solution used in this study contains a homogenous population of Aβ monomers (Giuffrida *et al*., 2009), able to engage and activate IGF‐IRs from recombinant cells at the lowest concentration of 10 nm (Giuffrida *et al*., 2015).

Although synthetic Aβ monomers might oligomerize in the culture medium, spectroscopy techniques operating at single‐molecule sensitivity levels have predicted that Aβ stays monomeric up to at least 3 μm (Nag *et al*., 2011) (i.e., a concentration three times higher than the highest concentration of monomeric Aβ used in this study). Moreover, the slow kinetic of Aβ self‐association *in vitro* (Kusumoto *et al*., 1998) is not consistent with the timing of CREB phosphorylation. Lastly, the contribution of Aβ self‐assemblies to the observed effect is unlike as concentrations of Aβ oligomers as low as 100 nm were not able to promote CREB phosphorylation in neurons, but rather reduced it. However, Aβ oligomers are highly heterogeneous and instable, ranging from low‐n oligomers (dimers, trimers, tetramers) to higher order assemblies (MW > 50 kD) (Ferreira *et al*., 2015), as those used in the present study. As different oligomeric species might recruit distinct signaling mechanisms, we cannot predict the effect of very low concentrations of low‐n oligomers, which have been shown to be relevant for proper synaptic function (Puzzo *et al*., 2011).

The role of CREB in AD pathology has been highlighted by the observation that pCREB is decreased in the nuclear fraction of postmortem prefrontal cortex (PFC) of individuals with AD, as compared to age‐matched, cognitively normal controls (Bartolotti *et al*., 2016a,b). In the same study, the authors reported that pCREB expression in peripheral blood mononuclear cells paralleled pCREB expression in the PFC, proposing pCREB as a biomarker for cognitive function in AD. Whether or not peripheral pCREB levels could depend on circulating Aβ monomers remain to be established. Noteworthy, skeletal muscle cells and insulinoma cells, which express functional IGF‐IRs, respond to mAβ stimulation (Giuffrida *et al*., 2015).

Consistent with the demonstration that mAβ induced CREB activation in neurons, mAβ was found to increase the neuronal transcription of BDNF. This increased transcription was associated with a rise in BDNF secretion. It remains to determine whether BDNF levels are dynamically regulated by the activity‐dependent Aβ release. Such a regulation would be in agreement with the evidence that in our hands mAβ was able to increase BDNF transcription and pre‐proBDNF synthesis (i.e., the precursor protein synthesis) as early as 6h after neuronal treatment (not shown).

Aβ oligomers failed to induce the transactivating activity of CREB, and therefore, they did not affect BDNF transcription and release. The last two‐ones were assessed at a time preceding frank neuronal loss, suggesting that a selective oAβ‐induced downregulation of BDNF levels was not among the earliest signs of neurodegeneration. Although oAβ did not appear to modify CREB activity, it reduced the basal expression of pCREB. The discrepancy between CREB phosphorylation status and CREB transcriptional activity, observed in the case of Aβ oligomers and not with Aβ monomers, indicates that monomers and oligomers could not share the phosphorylating signaling required for CREB‐regulated BDNF expression. Accordingly, following a brief exposure to oAβ, we observed a decreased expression of pCREB, whereas basal expression levels of pAKT stayed unchanged. Hence, monomers and oligomers of Aβ likely impinge on different CREB phosphorylation pathways. To this regard, Aβ oligomers have been shown to bind the insulin receptor (Xie *et al*., 2002; Townsend *et al*., 2007) and to promote a state of insulin resistance through the ERK‐dependent phosphorylation of insulin receptor substrate‐1 (Zhang *et al*., 2015). Conversely, Aβ monomers do not interact with the insulin receptor (Giuffrida *et al*., 2015) and have no effects on the ERK pathway (Giuffrida *et al*., 2009). It has been suggested that the impaired insulin signaling provoked by Aβ oligomers enables the reentry of postmitotic neurons into the cell cycle (Norambuena *et al*., 2017), a seminal process in AD pathogenesis (Copani *et al*., 2006). Whether or not Aβ monomers can oppose to this process, by sustaining IGF‐1 signaling, remains to be determined. Present data suggest that, at least, monomers and oligomers exert distinct effects on CREB protein functions, and that a loss of functional monomers and a building‐up of toxic oligomers could operate coordinately in determining the CREB dysregulation observed in AD (Pugazhenthi *et al*., 2011; Bartolotti *et al*., 2016a,b).

## Concluding remarks

**Scheme 1:**
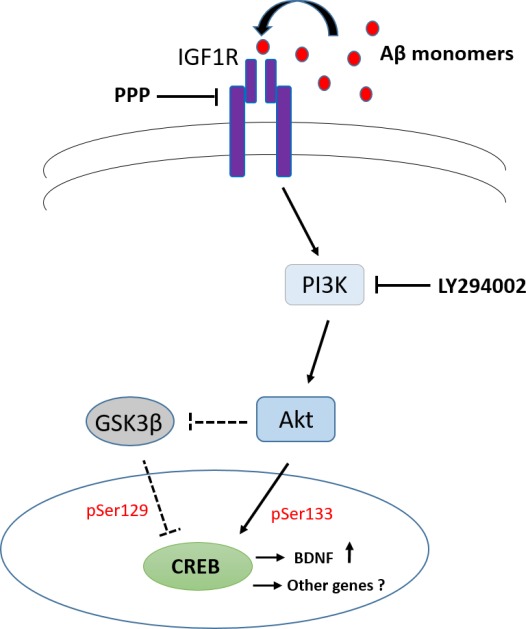
Schematic drawing of the interactions between Aβ(1‐42) and IGF‐I receptor signaling. Monomeric forms of Aβ activate type‐1 IGF receptors (IGF‐IRs), resulting in CREB phosphorylation (*via* PI3K activation) and BDNF transcription. Dashed lines refer to a possible, yet not proven, PI3K‐dependent pathway of CREB activation.

The results reported in this work strength the relevance of physiological Aβ. Aβ depletion is lethal to cultured neurons (Plant *et al*., 2003), and its blockade impairs learning and memory in adult mice (Morley *et al*., 2010; Puzzo *et al*., 2011, 2017). Low doses of synthetic Aβ ensemble mimic the memory enhancement effects of endogenous Aβ (Puzzo *et al*., 2008), likely *via* the regulation of transmitter release (Morley *et al*., 2010; Puzzo *et al*., 2011). Here we demonstrate that Aβ monomers are specifically able to activate CREB, a converging point for mechanisms and pathways involved in memory formation (Teich *et al*., 2015). By activating IGF‐IRs and the ensuing PI3K pathway, Aβ monomers regulate the expression levels of the CREB target gene, BDNF (Scheme 1), which is deeply involved in the regulation of cognitive functions (Budni *et al*., 2015). Our data suggest a new model whereby Aβ monomers may preserve cognitive decline.

## Experimental procedures

### Primary neuronal cultures

Animal care and experimentation were in accordance with institutional guidelines. Cultures of pure cortical neurons were obtained from rats at embryonic day 15 as previously described (Giuffrida *et al*., 2009). Cortical cells were dissected, mechanically dissociated, and seeded in Neural Q^™^ Basal Medium supplemented with GS21 (Globalstem, Rockville, Maryland, Stati Uniti). Cortical cells were plated on 24‐well plates precoated with 0.1 mg mL^−1^ poly‐D‐lysine and incubated at 37 °C with 5% CO_2_ in a humidified atmosphere. Cytosine arabinoside (1‐β‐D‐arabinofuranosylcytosine, Ara‐C) (3–10 μm) was added to the cultures 18 h after plating to avoid the proliferation of non‐neuronal elements and was kept for 3 days before medium replacement.

### Differentiated neuroblastoma SH‐SY5Y

The NB cell lines SH‐SY5Y were cultured in DMEM‐F12 (Gibco, Thermofisher, Waltham, MA, USA) supplemented with 10% heat‐inactivated fetal calf serum (Gibco, Thermofisher, Waltham, MA, USA), penicillin and streptomycin (100 mg mL^−1^ each), and 2 mm l‐glutamine at 37 °C, 5% CO_2_. Two weeks before the experiment, 5 × 10^3^ cells were plated on dishes with glass bottom in 5% fetal calf serum. Percentage of serum was gradually decreased until 1% of total. Five micromolar of all‐trans‐retinoic acid (RA) (SIGMA, Saint Louis, MO, USA) was used to promote neuronal differentiation and medium‐containing RA was changed every 3 days.

### Neuroglioma cell line, H4‐APPswe

The neuroglioma H4‐APPswe was cultured in Opti‐MEM (Gibco, Thermofisher) supplemented with 10% fetal calf serum (Gibco, Thermofisher), penicillin and streptomycin (100 mg mL^−1^ each), and 2 mm l‐glutamine at 37 °C, 5% CO_2_. To obtain the conditioned medium from this cell line, cells were seeded on 24‐well plates, and grown until confluence. Then, cells were washed twice with PBS buffer and medium was replaced with Neural Q basal Medium supplemented with penicillin and streptomycin (100 mg mL^−1^ each), 2 mm l‐glutamine, and 2% GS21 (Globalstem). To allow medium enrichment of endogenous Aβ, cells were maintained for 6 and 24 h, in the presence or in absence of β‐secretase inhibitor (5 μm) or γ‐ secretase inhibitor (1 μm) (Calbiochem). Then, medium was collected and immediately assessed for its concentration of Aβ. The remaining part was briefly stored at −80 °C. The 24h conditioned medium was subsequently used to treat mature cortical neurons. When required, synthetic Aβ monomers (100 nm) were added to rescue the lack of endogenous Aβ (β‐/γ‐secretase Inhibitor CM).

### Aβ peptides preparation

Aβ 1‐42 (HFIP‐treated) was purchased from Bachem Distribution Services GmbH, Germany, dissolved at a 5 mm concentration in anhydrous dimethyl sulfoxide (DMSO) and stored at −20 °C. At the time of its use, a solution of 100 μm Aβ in ice‐cold DMEM F‐12 was prepared and allowed to oligomerize overnight at 4 °C according to the Lambert protocol (Lambert *et al*., 1998) and our previously described method (Giuffrida *et al*., 2009). Monomers and oligomers (>50 kD) were isolated from the peptide suspension by filtration through cutoff filters as previously described (Giuffrida *et al*., 2009).

### MTT assay

Cortical cells were seeded in 24‐well plates precoated with poly‐d‐lysine (0.1 mg mL^−1^) and incubated at 37 °C with CO_2_ (5%) under a humidified atmosphere; 18h after plating, cytosine β‐d‐arabinofuranoside ((3–10 μm)) was added to the cultures to avoid the proliferation of glial cells. Mature neuronal cultures between 6 and 8 days *in vitro* were treated with mAβ 100 nm and two different concentrations of oAβ (100 nm and 2 μm) for 24 and 48h.

Cell viability was assessed by the 3‐[4,5‐dimethylthioazol‐2‐yl]‐2,5‐diphenyl tetrazolium bromide (MTT) assay. Cultures were incubated with MTT (0.9 mg mL^−1^) for 2h at 37 °C, then lysed by incubation with DMSO for 15 min at 37 °C. Formazan production was evaluated in a plate reader (absorbance = 570 nm).

### Western blot analysis

Primary neuronal cultures, treated with mAβ1‐42 (100 nm‐1 μm), IGF‐1 (5 ng mL^−1^), oAβ1‐42 (100 nm and 2 μm) or forskolin (30 μm) for 30 min in PBS, were harvested in RIPA lysis buffer (Alfa Aesar, GmbH Co KG) containing protease and phosphatase inhibitors cocktail mixes (both from Pierce‐Thermofisher). When required, a 30’ pretreatment with LY294002 (10 μm) or picropodophyllin (PPP) (500 nm) (both from Calbiochem) was used.

Protein concentration was determined by BCA protein Assay Kit (Pierce‐Thermofisher). Equal amounts of proteins (30 μg) were separated by sodium dodecyl sulfate–polyacrylamide gel electrophoresis (SDS‐PAGE) on precast 4–12% gradient gels (Bolt, Invitrogen, Thermofisher) and electro‐transferred onto a nitrocellulose membrane in a wet transfer cell. Nonspecific binding was prevented by incubation with blocking buffer (LiCor, Biosciences). Membranes were incubated overnight at 4 °C with the following primary antibodies: p(ser 133)‐CREB, CREB, p(ser 473)‐Akt, and pan AKT (all used at 1:1000 dilution, Cell signaling Technology, Beverly, MA, US). Secondary goat anti‐rabbit labeled with IR dye 680 (1:25.000 Li‐COR 280 Biosciences, Lincoln, Nebraska, US) and goat anti‐rabbit labeled with IRdye 800 (1:20.000 Li‐COR Biosciences, Lincoln, Nebraska, US) were used at RT for 45 min. Hybridization signals were detected with the Odyssey CLx Infrared Imaging System (LI‐COR Biosciences Lincoln, Nebraska, US).

Characterization of H4‐APPswe conditioned medium (CM): Aliquots (5 μL) of 24 and 48h conditioned medium of H4‐APPswe were diluted into a 4× Bolt LDS sample buffer without reducing agents and loaded into a precast Bolt Bis·Tris gels (4–12%, Life Technologies, Carlsbad, California, US) running in 2‐morpholin‐4‐ylethanesulfonic acid (MES) buffer. Proteins were transferred onto a nitrocellulose membrane in a wet transfer cell (Mini Blot Module Life Technologies, Carlsbad, California, US), blocked in Odyssey blocking buffer (Li‐COR Biosciences, Lincoln, Nebraska, US), and then incubated at 4 °C overnight with 1:1000 mAb 6E10 (Covance, Princeton, New Jersey, US) in Odyssey blocking buffer/PBS‐Tween 1:1. Anti‐mouse secondary antibody, labeled with IR dye 800 (1:20000 Li‐COR Biosciences, Lincoln, Nebraska, US), was used at RT for 45 min. Hybridization signals were detected with the Odyssey infrared imaging system (LI‐COR Biosciences, Lincoln, Nebraska, US).

### Indirect pCREB immunofluorescence analysis

To confirm CREB phosphorylation and to prove its nuclear localization after Aβ monomer treatment, SH‐SY5Y were seeded in 35‐mm glass bottom dishes and differentiated with RA as described in the cell culture section. To perform the experiment, cells were washed and stimulated for 30 min with monomeric Aβ1‐42 (100 nm), forskolin (30 μm), or oligomeric Aβ1‐42 (100 nm) in PBS buffer. Cells were then fixed in 2% formaldehyde and permeabilized using 0.1% Triton X‐100. Unspecific binding was blocked by 30 min of incubation in 4% bovine serum albumin (BSA) in 0.1% Triton X‐100‐PBS. pCREB was detected by incubating overnight cells with rabbit anti‐p (ser‐133)CREB antibody (1:500, Cell signaling). After PBS washing, cells were exposed for 1 h at RT to the secondary antibody (anti‐rabbit Texas Red conjugated). Hoechst (Molecular Probes, 1 μg μL^−1^) was used to stain nuclear DNA. Images were analyzed under a Leica DMI 6000B epifluorescence inverted microscope with Adaptive Focus Control. Sixty red (pCREB)/blue (Hoechst) ratio values were taken from twenty nuclear ROI (region of interest)/microscopic field, in 3 fields/dish.

### Real‐time PCR

Total RNA was extracted from primary cortical neurons using TRIzol reagent (Invitrogen, Thermofisher), following the manufacturer instructions. cDNA was synthesized from 1 μg of total RNA using High Capacity cDNA Reverse Transcription kit (Applied, Thermofisher) with the oligo(dT) primers. Quantitative real‐time PCR for the expression of BDNF was performed using SYBR Green Master Mix with the following primers: BDNF‐Forward 5′‐TCAAGCTGG AAGCCTGAATGAA‐3′, BDNF‐reverse 5′‐CCC AGT CAG GTA ACC ACT AAC AC‐3′. GAPDH was used as endogenous controls for normalization (GAPDH‐fw 5′‐GAACATCATCCCTGCATCCA‐3′, GAPDH‐rv 5′‐CCAGTGAGCTTCCCGTTCA‐3′; Amplification reactions were performed on an ABI Prism 7500 (PE Applied Biosystems) according to the manufacturer's instructions. Relative expression levels of the tested gene were determined using the 2^−ΔΔCt^.

### CREB DNA binding assay

Primary neuronal cultures were treated with two different concentrations of mAβ (10 and 100 nm) for 30 min in PBS. 100 nm Aβ oligomers and 30 μm forskolin were used as negative and positive control, respectively. After treatments, neurons were harvested in RIPA lysis buffer (Alfa Aesar, GmbH Co KG) containing protease and phosphatase inhibitors cocktail mixes (both from Pierce‐Thermofisher). Protein concentration was calculated using BCA protein assay kit (Pierce‐Thermofisher), and an equal amount of proteins (15 μg) from each sample was tested. The DNA binding activity was measured by CREB(pSer133) Transcription factor Assay kit (Cayman CHEMICAL, Ann Arbor, MI, USA) in whole cell lysates, following the manufacturer's instruction.

### Enzyme‐linked immuno sorbent assay (ELISA)

Levels of Aβ42 in conditioned medium of H4‐APPswe were determined with the Human/Rat beta‐Amyloid (42) ELISA Kit, High Sensitivity from Wako Chemicals USA, Inc. Medium‐containing human recombinant Aβ42, collected at 6 and 20 h incubation, was diluted with the provided sample buffer immediately prior to the assay. Levels of BDNF in neuronal culture supernatants were measured with Rat BDNF ELISA kit Thermofisher.

## Funding

This work has been supported by grant SIR RBS1148TJD from MIUR (Italian Ministry for Research and University) to Maria Laura Giuffrida. Stefania Zimbone has been supported by a SIR project postdoctoral contract.

## Author contributions

E.R., M.L.G., and A.C. were responsible for the design, acquisition, analysis, interpretation, preparation, and approval of manuscript draft. S.Z., M.L.G, I.M., F.G., and G.P. performed experiments, analyzed the data, made the figures, and approved the final version of the manuscript.

## Conflict of interests

The authors declare that they have not conflict of interests.
